# Manganese‐Induced Production of Antioxidant Polyene Steroids in the Extremophile Fungus *Talaromyces fuscoviridis* Isolated From Copper‐Mine Soil

**DOI:** 10.1002/cbdv.202501816

**Published:** 2025-11-21

**Authors:** Mauricio Augusto Pinto Moreno da Silva Alves, Alef dos Santos, Eduardo Jorge Pilau, Pedro Henrique de Oliveira Santiago, Javier Alcides Ellena, Marilene Nunes Oliveira, Edson Rodrigues Filho

**Affiliations:** ^1^ Laboratory of Micromolecular Biochemistry of Microorganisms, Department of Chemistry Federal University of São Carlos São Carlos São Paulo Brazil; ^2^ Department of Chemistry Umeå University Umeå Sweden; ^3^ Laboratory of Biomolecules and Mass Spectrometry, Department of Chemistry State University of Maringá Maringá Paraná Brazil; ^4^ Institute of Physics of São Carlos University of São Paulo São Carlos São Paulo Brazil; ^5^ Institute of Exact Sciences Federal University of the South and Southeast of Pará Marabá Pará Brazil

**Keywords:** antioxidant activity, bioprospecting, molecular networking, untargeted metabolomics

## Abstract

The fungus *Talaromyces fuscoviridis* was isolated from soil collected in a copper‐mine tailings basin in Pará State, Brazil, an extreme environment that can induce the production of bioactive secondary metabolites. Extracts and fractions obtained after cultivation were analyzed by liquid chromatography coupled to high‐resolution mass spectrometry (LC–HRMS) and compared using Global Natural Products Social molecular networking (GNPS), which revealed a family of seven putative polyunsaturated steroids. Isolation led to the characterization of two major steroids: the known Ergosta‐3(4),5(6),7(8),14(15),22(23)‐pentaene (1a) and a previously unreported metabolite, Ergosta‐5(6),7(8),14(15),22(23)‐tetraen‐3‐ol (2a). Their structures were elucidated through comprehensive spectroscopic and spectrometric methods (e.g., UV–Vis, NMR, and high‐resolution mass spectrometric [HRMS]), with the structure of steroid 2a being unequivocally confirmed by single‐crystal x‐ray diffraction (SCXRD). Notably, the biosynthesis of these steroids was significantly enhanced when the cultivation medium was supplemented with manganese chloride (MnCl_2_). Furthermore, the steroids were tested for their in vitro antioxidant activity against the 2,2‐diphenyl‐1‐picrylhydrazyl (DPPH) free radical using an adapted UHPLC–DAD method, showing over 80% of the scavenging capacity of β‐carotene at the lowest tested concentrations.

## Graphical abstract

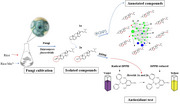

## 1 Introduction

Although fungi generally exhibit a prolific secondary metabolism, yielding many important molecules useful in biotechnological applications for human well‐being [1, 2, 3], the diversity of fungi does not always correlate with the diversity of fungal natural products. This is likely due to the fact that while microorganisms possess a large number of distinct biosynthetic gene clusters (BGCs) encoded in their genomes, the expression of this genetic machinery depends on multiple external factors that are often unknown or difficult to determine [ 4, 5, 6, 7]. As a result, researchers worldwide often re‐isolate already known fungal natural products.

This realization led many natural product chemists, in conjunction with molecular biologists, to hypothesize that microorganisms living in extreme or stress‐inducing environments may activate different BGCs in order to adapt to inhospitable substrates [8, 9]. Consequently, the fungal biosphere present in potentially harmful environments has received special attention from the scientific community, which seeks to understand the mechanisms these microorganisms develop to adapt to challenging conditions and how this affects their metabolism. Such studies provide valuable contributions to the discovery of new targets for biotechnological applications and drug discovery [10, 11, 12].

Studies have shown that volcanoes [13, 14], different types of caves [15, 16, 17], highly saline lakes [18], and the deep ocean [19] constitute promising sources of microorganisms for chemical and bioprospecting studies. Brazil comprises numerous environments that impose severe physicochemical constraints on microbial colonization and growth [20]. Such extreme habitats represent valuable natural laboratories for exploring microbial adaptation and the biosynthesis of structurally diverse, biologically active metabolites.

The present study focuses on extremotolerant fungal isolates obtained from metal‐rich substrates, particularly from copper‐mine soils located in Pará State, northern Brazil. During a recent sampling campaign, several mining sites were surveyed in search of cultivable fungal strains. Soil collected from a copper‐mine tailings basin yielded a limited number of morphologically distinct isolates, which were initially characterized on the basis of their macro‐ and micromorphological features under optical microscopy and subsequently identified through molecular taxonomic analyses [21].

Among the isolates obtained, *Talaromyces fuscoviridis* [21] was selected for an in‐depth chemical and functional investigation aimed at elucidating its metabolic profile and assessing its biological activities. Considering the ability of this fungus to grow in copper‐enriched substrates, its behavior was also investigated under manganese supplementation, another environmentally relevant transition‐metal cation, to explore potential relationships between these elements in the regulation of secondary metabolism. For the first time, untargeted metabolomic approaches integrated with molecular network analyses were employed to characterize the secondary metabolites produced by *Talaromyces fuscoviridis* and to evaluate the effect of manganese on their metabolism.

## 2 Materials and Methods

### 2.1 General Experimental Procedures

The ^1^H and ^13^C NMR spectra were acquired in CDCl_3_ solution using a Bruker Avance III (9.4 Tesla) instrument, equipped with an autosampler, a 5 mm ATMAR BFO probe head, and a *z*‐axis field gradient, operating at 400 and 100 MHz, respectively. Tetramethylsilane (TMS) served as the internal reference signal. High‐resolution mass spectrometric (HRMS) data were acquired on a hybrid quadrupole‐time‐of‐flight mass spectrometer (QToF MS, Impac II, Bruker Daltonics, Bremen, Germany) coupled to an ultra‐high performance liquid chromatography (UHPLC) (Shimadzu Nexera X2, Tokyo, Japan). Separation was performed using a reversed‐phase C‐18 column (2.1 × 100 mm^2^, 1.7 µm particle size, 135 Å porous; Waters, Billerica, MA, USA). Gas chromatography (GC–MS) analyses were carried out using a QP‐2010‐plus mass spectrometer coupled to a GC‐17A gas chromatograph (Shimadzu, Tokyo, Japan), equipped with a Restek‐Rxi‐5 ms fused silica column (10 m × 0.10 mm × 0.10 µm; J&W Scientific). Preparative medium‐pressure liquid chromatography (MPLC) was performed using a Teledyne‐Isco Combiflash RF 200 system, employing RediSep gold silica gel packed columns. Thin‐layer chromatography (TLC) was carried out on pre‐coated silica gel GF254 plates with alumina backing; spots were visualized under UV light or by spraying with 10% sulfuric acid in EtOH followed by heating. The antioxidant activity assays were conducted using UHPLC system (Acquity M‐Class, Waters, Manchester, UK) equipped with a C‐18 column (2.1 × 100 mm^2^, 1.7 µm). Single‐crystal x‐ray diffraction (SCXRD) data were collected at 100 K on a Rigaku XtaLAB Synergy‐S Dualflex diffractometer equipped with a Hypix‐6000 HE detector, using Cu*K*_α_ radiation (*λ* = 1.54184 Å). Gene sequencing for fungal identification was performed using an ABI 3500XL Series automatic sequencer (Applied Biosystems).

The organic solvents dichloromethane, ethyl acetate, methanol, and ethanol were of analytical grade and obtained from Synth (Diadema, SP, Brazil) and Quimis (Indaiatuba, SP, Brazil). Chromatographic grade acetonitrile and methanol (Panreac, Barcelona, Spain) were used for HPLC separations. All other chemicals were purchased from Sigma‐Aldrich Chemical and used without further purification.

### 2.2 Fungus Isolation

Soil samples were collected from the mining waste basin of a copper mine in Pará State, Brazil, at a depth of 0–10 cm (coordinates: −6.429729, −50.081160). A soil suspension was prepared by adding 25 g of soil to 225 mL of sterilized deionized water. Aliquots (50 mL) of this suspension were spread onto potato dextrose agar (PDA) plates, which were supplemented with chloramphenicol and tetracycline (10 mg/L each) to suppress bacterial growth. Developing fungal colonies were purified by successive subculturing onto fresh PDA plates. The pure strain was preserved in sterilized water and initially deposited in the Microbial Collection of the Multidisciplinary Biology Laboratory at the Federal University of South and Southeast of Pará (UNIFESSPA). It was subsequently shared with the Microbial Collection of the Laboratory of Micromolecular Biochemistry of Microorganisms (LaBioMMi), Department of Chemistry at the Federal University of São Carlos (Campus São Carlos, Brazil). The isolated strain, *Talaromyces fuscoviridis*, was assigned the accession code P_3_W_1_M_2_.

### 2.3 Fungus Identification

For morphological observations, the fungus was cultivated in microscale using slide culture. In this method, a suspension of fungus cells was inoculated onto microscopy slides containing 40 µL of PDA culture medium. The prepared suspension was transferred to the culture medium using a platinum loop previously sterilized by flame. Following inoculation, a cover slip was carefully placed over the surface of the culture medium. The entire procedure was conducted in a sterile environment.

To maintain suitable cultivation conditions, a piece of moistened cotton was placed inside the Petri dish to preserve humidity, ensuring that it did not come into contact with the slide or the cover slip. The culture plate was then sealed and incubated at a temperature of 27°C for 6 days in a biochemical oxygen demand (BOD‐type incubator). After the incubation period, the slides were examined under a bright‐field optical microscope, allowing for the visualization of morphological aspects of the observed fungal structures.

Partial sequencing of the β‐tubulin gene was performed to confirm the molecular identification of the fungal isolate. Mycelial biomass grown on PDA was used for genomic DNA extraction following the phenol–chloroform–isoamyl alcohol method. The β‐tubulin gene region was amplified by polymerase chain reaction (PCR) using the primer pair Bt2a/Bt2b. The PCR product was purified with the GFX PCR DNA and Gel Band Purification Kit (GE Healthcare) and directly sequenced on an ABI 3500XL Series automatic sequencer.

The resulting partial sequences were assembled into a consensus sequence by combining overlapping fragments and compared with reference sequences available in the GenBank and CBS databases. Sequence alignment was performed with CLUSTAL X integrated into BioEdit 7.2.6. Phylogenetic analysis was conducted in MEGA v11.0 using the Kimura two‐parameter model [22], and a neighbor‐joining tree was constructed with bootstrap support values calculated from 1000 replicates.

### 2.4 Fungus Cultivation, Extract Production, and Fractionation

The fungal isolate was initially cultured on PDA medium, which had been sterilized in a Phoenix vertical autoclave at 121°C under 1 atm for 15 min. After sterilization, the plates were cooled to room temperature (25°C). The plates were inoculated in a laminar flow environment using 5 mm diameter fragments of the *Talaromyces fuscoviridis* isolate and incubated for 10 days.

A spore suspension was prepared by transferring fungal cells from the plates into a Falcon tube containing 30 mL of autoclaved distilled water using a platinum loop. The suspension was homogenized by agitation on a vortex mixer. Spore enumeration was performed by microscopic counting using a Neubauer counting chamber (depth: 0.100 mm; smallest grid area: 0.0025 mm^2^). This instrument, from the Global Optics brand, features dimensions (*L* × *W* × *H*) (7.5 cm × 3.2 cm × 0.35 cm). The concentration of the spore suspension was subsequently standardized to 1.0 × 10^6^ cells/mL.

The standardized spore suspension was distributed among 10 of 1000 mL Erlenmeyer flasks, each containing 200 g of rice that had been previously autoclaved twice at 121°C under 1 atm for 15 min. The solid cultures were then incubated under static conditions at 28°C for 30 days.

Following the incubation period, the fungal biomass was subjected to a sequential solvent extraction aiming to optimize the recovery of bioactive secondary metabolites. Ethanol extraction (pre‐treatment): Initially, the cultures were extracted with approximately 200 mL of ethanol per flask. This initial extraction was performed primarily to remove most of the residual water from the culture medium. This pre‐treatment step is crucial to prevent the formation of emulsions and phase separation issues during the subsequent extraction with ethyl acetate, a less polar solvent. Ethyl acetate extraction: After the ethanolic solvent was removed, the residual biomass was subjected to extraction with ethyl acetate, aiming for the optimized recovery of lipophilic bioactive compounds. The mechanical procedure was equivalent in both solvent steps: The biomass was macerated with a glass rod to disaggregate the mycelium and maximize the contact surface, followed by sonication in an ultrasonic bath for 20 min.

The final combined extract was filtered under vacuum, and the organic phase was transferred to a round‐bottom flask for solvent evaporation. This extraction and work‐up procedure was repeated three times. An aliquot of the crude extract was used for metabolomic profile analysis by liquid chromatography coupled to high‐resolution mass spectrometry (LC–HRMS) using a Bruker QToF instrument.

The crude extract was initially fractionated by dry column vacuum chromatography (DCVC) on a silica gel 60 column (mesh 70–230; 30 cm height × 4.0 cm diameter). Elution was carried out with mixtures of hexane, ethyl acetate, and methanol in increasing polarity, yielding six fractions (Fr1–Fr6) as follows: Fr1, 95:5:0; Fr2, 70:30:0; Fr3, 0:100:0; Fr4, 0:90:10; Fr5, 0:70:30; and Fr6, 0:0:100 (v/v/v). For each elution step, a total solvent volume of 600 mL was used.

Fraction Fr3, which exhibited the richest steroidal profile, was selected for further purification. A total of 145.0 mg of this fraction was subjected to MPLC on a silica gel column using a Combiflash RF 200 system equipped with a RediSep 200 g cartridge. The mobile phase consisted of a hexane–ethyl acetate gradient, starting with 5% hexane and 95% ethyl acetate for initial column conditioning. The gradient was subsequently increased to 100% ethyl acetate over the course of the run, allowing clear resolution and isolation of the individual steroidal peaks. Chromatographic separation was performed at a flow rate of 8 mL/min, with monitoring at 254 and 310 nm, and a total run time of 55 min. The major chromatographic fractions afforded crystalline material upon slow solvent evaporation. After complete solvent removal, the crystals were collected and subjected to structural characterization, which ultimately yielded two pure steroids: compound **1**, obtained as an amorphous white solid, and compound **2**, obtained as colorless needles.

### 2.5 UHPLC–MS/MS Analysis of Fungal Extracts

The metabolomic profile analysis of the crude extracts was conducted using UHPLC system (Shimadzu) coupled to a high‐resolution mass spectrometer (QToF, Bruker). Chromatographic separation was achieved on a C‐18 column (from Waters). The mobile phase consisted of water with 0.1% formic acid (solvent A) and acetonitrile with 0.1% formic acid (solvent B). A gradient elution was applied starting with 95% A and 5% B for 1 min, changing to 30% A and 70% B at 12 min, reaching 2% A and 98% B at 20 min, and returning to initial conditions between 20 and 25 min, ensuring column re‐equilibration. The flow rate was maintained at 0.25 mL/min and a column temperature at 40°C.

Prior to analysis, the mass spectrometer was calibrated using a sodium formate solution (10 mmol/L; isopropanol:water, 1:1 v/v) with an addition of 50 µL of concentrated formic acid. During operation, the electrospray ionization (ESI) ion source was set in positive ionization mode, with a capillary voltage of 4500 V and a final plate offset of −500 V. The dry gas was maintained at 8 L/min and 180°C, and the nebulizer gas was adjusted to 4 bar. Data acquisition ranged from *m/z* 50 to 1800 at a rate of 5 Hz. For tandem MS/MS analysis, an automatic scan fragmentation MS/MS was performed targeting the five most intense ions from each chromatographic peak, optimizing data quality and ion‐specific fragmentation patterns for subsequent metabolite identification.

The mass spectral data‐based molecular network was established using the Global Natural Products Social Molecular Networking (GNPS) platform (http://gnps.ucsd.edu). High‐resolution MS data from the samples were acquired using an ESI source, which usually provides prominent precursor ions. Then the LC–MS/MS data were analyzed and processed using MS‐DIAL software version 5.5, with mass detection assessed within a retention time range of 0.3–25.0 min, utilizing centroid mass detection and a noise level threshold of 1.0 × 10^4^. Tolerance parameters for both MS1 and MS2 were set in accordance with GNPS standards, with MS1 at 0.02 Da and MS2 at 0.02 Da. The mass range was set from *m/z* 50 to 1500. Deconvolution parameters included a window of 0.5 and an MS/MS cutoff abundance at amplitude level 30. Alignment was based on reference data applied to a blank, with a retention time tolerance of 0.5 min. Both the retention factor and MS1 factor also used a value of 0.5, applicable within a reference range of 0–1.

Files were processed through the spectral networks algorithm (GNPS) in three formats: an *mgf* file of deconvoluted spectra by MS‐DIAL 5.5, a quantification table of the peaks generated in this process, and a metadata table with sample information such as sample type and experimental mode. In GNPS, the precursor ion mass tolerance was 0.02 Da, a fragment ion mass tolerance was 0.02 Da, a minimum of five matching peaks was used, and a score threshold of 0.7. Advanced search options included a library class with a history higher than 1 per spectrum and the use of the GNPS spectral library. Advanced network options were set to a minimum cosine pair of 0.65 and a TopK network of 10. Network visualization was performed using Cytoscape software version 3.10.2.

### 2.6 Fungus Cultivation in the Presence of MnCl_2_·4H_2_O

To evaluate the influence of the manganese cation on metabolite production, the fungus *Talaromyces fuscoviridis* was cultivated on modified rice, prepared with the addition of MnCl_2_·4H_2_O at a final concentration of 125 mmol/L. The cultivation was conducted in triplicate under the same conditions as the control medium (rice hydrated only with distilled water), using 250 mL Erlenmeyer flasks containing approximately 30 g of rice and 60 mL of the corresponding solution. After sterilization, each Erlenmeyer flask was aseptically inoculated with 1 mL of a spore suspension standardized to 1 × 10^6^ colony‐forming units (CFU)/mL. The inoculated cultures were subsequently incubated under static conditions at 25°C for 20 days, allowing for the development of mature mycelial biomass and metabolite accumulation. At the end of the cultivation period, ethanolic extracts were obtained by static maceration and concentrated using a rotary evaporator. Subsequently, the extracts were analyzed by LC–MS/MS to qualitatively and quantitatively compare the metabolites produced. The comparative analysis was based on molecular networking (GNPS) and relative quantification through peak area extraction of the annotated compounds.

### 2.7 GC–MS Analysis

An aliquot of the isolated substance was analyzed by fast GC–MS (Shimadzu QP‐2010‐plus) equipped with a 10 m × 0.10 mm × 0.10 µm Restek‐Rxi‐5 ms fused silica column. Helium was used as the carrier gas at a linear velocity of 42 cm/s. The injector temperature was set at 250°C. Initially, the column was held at 70°C for 1 min, then increased at a rate of 20°C/min up to 320°C, and maintained at this temperature for 2 min. The total analysis time was 15 min with a 1 µL injection volume in splitless mode. The EI ion source temperature was fixed at 240°C and the interface temperature at 250°C. Ion scanning was operated between *m/z* 70 and 670, and acquired from 3 to 15 min. This method was developed specifically for this study.

### 2.8 Single‐Crystal X‐Ray Diffraction

The slow evaporation of a methanolic solution of steroid **2** at room conditions yielded needle‐like crystals suitable for SCXRD analysis. The SCXRD data were collected at 100 K on a Dualflex diffractometer using a Cu*K*_α_ radiation. The CrysAlisPro program was used for the data collection, cell refinement, data reduction, and processing. The structure was solved using the intrinsic phasing method from the SHELXT program. All non‐hydrogen atoms were found in the Fourier map and subsequently refined by the nonlinear least‐squares minimization on *F* ^2^ method using the SHELXL program considering anisotropic parameters. Hydrogen atoms were stereochemically positioned using the riding model. The Olex2 program was used for the solution and refinement of the structure. The graphical illustrations were obtained using the Mercury. Selected information about the data collection and refinement are listed in Table S1. The CIF file of steroid **2** was deposited in the Cambridge Structural Database with CCDC number 2314479. Copies of the data can be obtained free of charge from www.ccdc.cam.ac.uk.

### 2.9 Antioxidant Potential Assay Against 2,2‐Diphenyl‐1‐Picrylhydrazyl (DPPH)

The radical scavenging activity of reference compounds, isolated steroids and fractions was determined by measuring the ratio DPPH/DPP^•^ after mixing the samples with a methanol:acetone solution of DPPH at a concentration of 0.075 mM in separate glass vials. The evaluation was performed after a reaction time of 30 min in a dark environment at a temperature of 25°C. Stock solutions of reference and isolated compounds were prepared at concentrations of (2.0, 1.0, 0.5, 0.25, 0.125, 0.0625 mg/mL) in a methanol:acetone (1:1) v/v solution, respectively. The stock solutions were stored in the dark at a temperature of 5°C.

For the antioxidant test, β‐carotene with high analytical grade was used as a reference compound. The DPPH/DPP^•^ ratio was measured by reverse phase chromatographic analysis using an UHPLC (Waters—Acquity) and a C‐18 column eluted with isocratic mobile phase composed of 30% water and 70% acetonitrile during 6 min at a flow rate of 0.4 mL/min, injection volume of 5.0 µL, column at 25°C and detection at 330 nm.

The antioxidant activity of the samples was calculated using the following equation [23]:

$$\%\mathrm{DPPH}\mathrm{or}\mathrm{DP}P^{{•}^{\cdot }}\mathrm{peak}\mathrm{area}\mathrm{reduction}=\left(\left(\mathrm{Aaa}-\mathrm{Ada}\right)/\mathrm{Aaa}\right)\times 100$$

where Aaa is the peak area of DPPH or DPP^•^ before addition of the antioxidant, and Ada is the peak area of DPPH/DPP^•^ after addition of the antioxidant.

## 3 Result and Discussion

### 3.1 Identification of the Fungus as *Talaromyces fuscoviridis*

**FIGURE 1:**
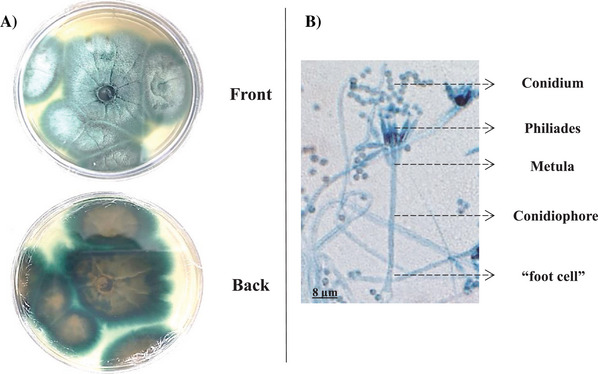
Morphological characteristics of *Talaromyces fuscoviridis*. (A) Colony morphology on PDA plates after 10 days of incubation at 27°C, showing blue‐green pigmentation on the surface and yellowish reverse coloration. (B) Micrograph obtained from slide culture under bright‐field microscopy (40×), highlighting the verticillate conidiophores with metulae and phialides producing chains of conidia. Scale bar = 8 µm.

The fungus grew in PDA Petri dishes, forming intense blue‐greenish pigmented structures visible at the front of the plate and yellowish at the back. The micrograph (Figure 1) revealed verticillate conidiophores with the formation of phialides, and the conidia were organized in chains, with a higher proportion in the upper areas. In the micrograph, it was possible to observe that this fungus has a base in the shape of a “T” or “L,” commonly referred to as a “foot cell.” The conidiophore extends from the foot cell, and the conidiogenic cells, metulae, and phialides are formed from it. These characteristics are well compatible with fungus in *Talaromyces* genus [21].

**FIGURE 2:**
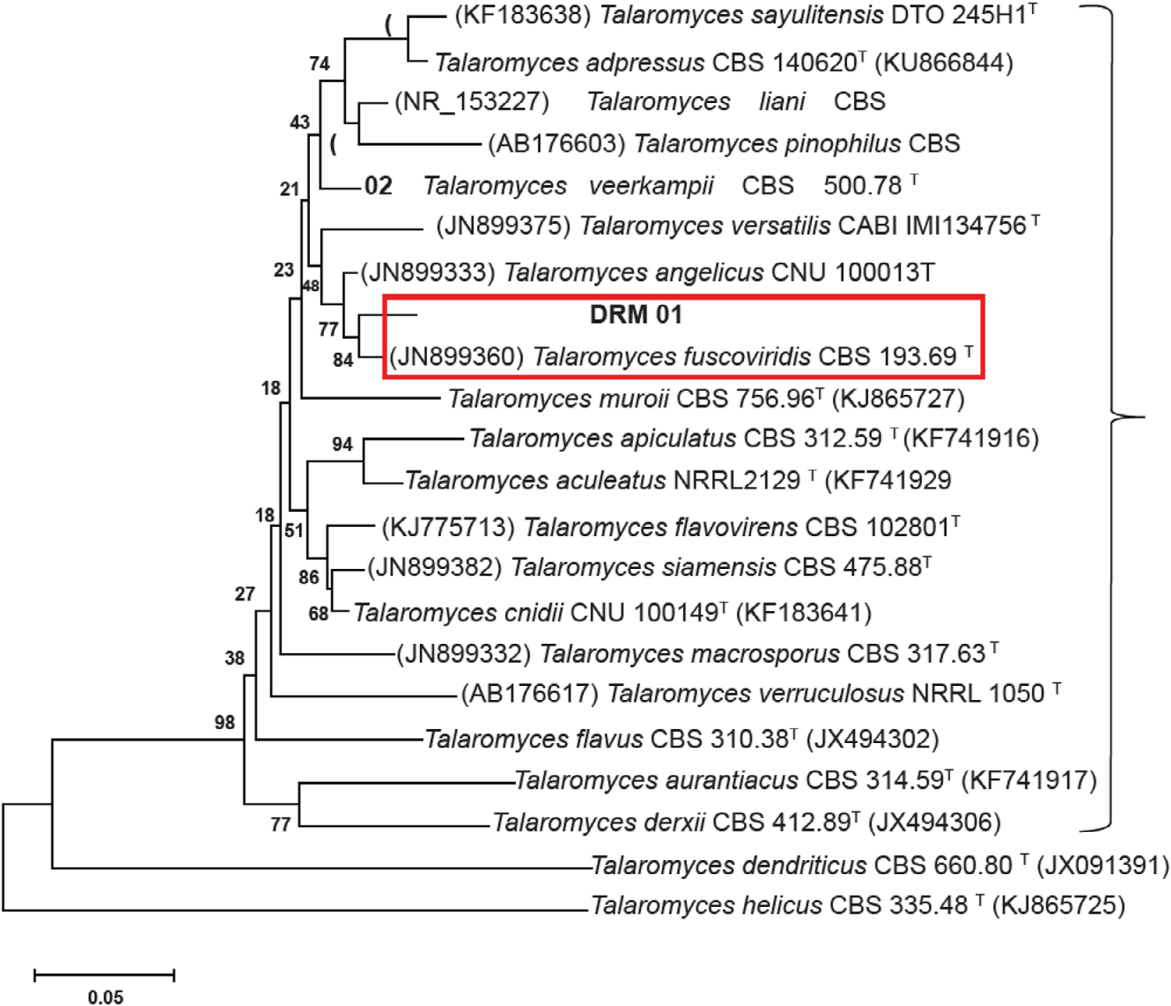
Dendrogram based on genetic distance using the neighbor‐joining method, demonstrating the relationship between the partial sequence of the β‐Tub region of the microbial sample and sequences of strains of related microorganisms present in the Mycobank databases (CBS Knaw, current Westerdijk Fungal Biodiversity Institute) and Genbank.

Although the fungus genus was clear by morphological aspects inspection, the species was determined only by genomic DNA sequencing. The β‐Tub region sequence of the sample showed 95%–98% similarity to sequences from the same ribosomal region of different species within the *Talaromyces* genus, as deposited in the GenBank and CBS‐Knaw databases. Genetic distance analysis placed the fungus sample in a cluster within the *Talaromyces* section, closely related to the *Talaromyces fuscoviridis* CBS193.69 type strain. Consequently, the results from the database analyses and phylogeny suggested that the final identification of the fungus is *Talaromyces fuscoviridis* (Figure 2).

### 3.2 Metabolite Profile of *Talaromyces fuscoviridis*

The molecular networks approach was used to draw the metabolite profile of the extract obtained from the fungus *Talaromyces fuscoviridis*. This approach allowed the comparison of MS/MS data, their organization in families of molecular structures, and visualization. These metabolome analyses are based on integrated LC–MS/MS analysis with various metabolomic libraries (NIST, NPATLAS, MONA, MassBank, METLIN) and the GNPS, as well as in silico fragmentation algorithms such as SIRIUS and MS‐FINDER [24, 25, 26].

**FIGURE 3:**
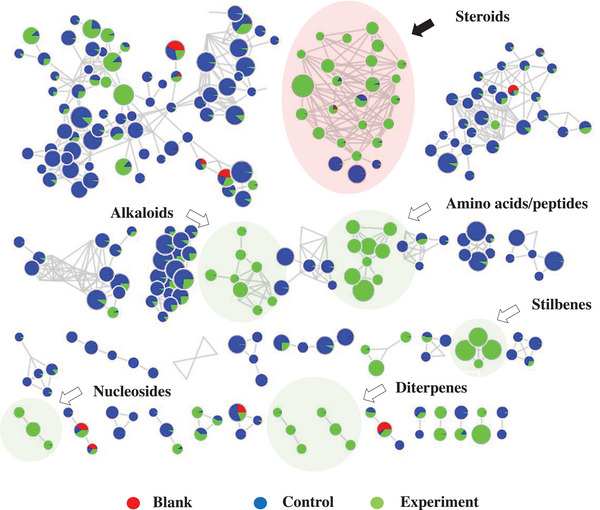
Molecular network obtained from the fungal extract of *Talaromyces fuscoviridis* that was grouped by the Cytoscape 3.10.2 software, emphasizing the main annotations. The steroid cluster stands out in red.

The molecular network obtained provided a total of 312 clusters of nodes interconnected by 752 edges. It was possible to annotate various classes of secondary metabolites that were grouped into different clusters, and also primary metabolites such as amino acids and peptides (Figure 3). The cluster of compounds presenting a steroidal skeleton is highlighted in red.

**FIGURE 4:**
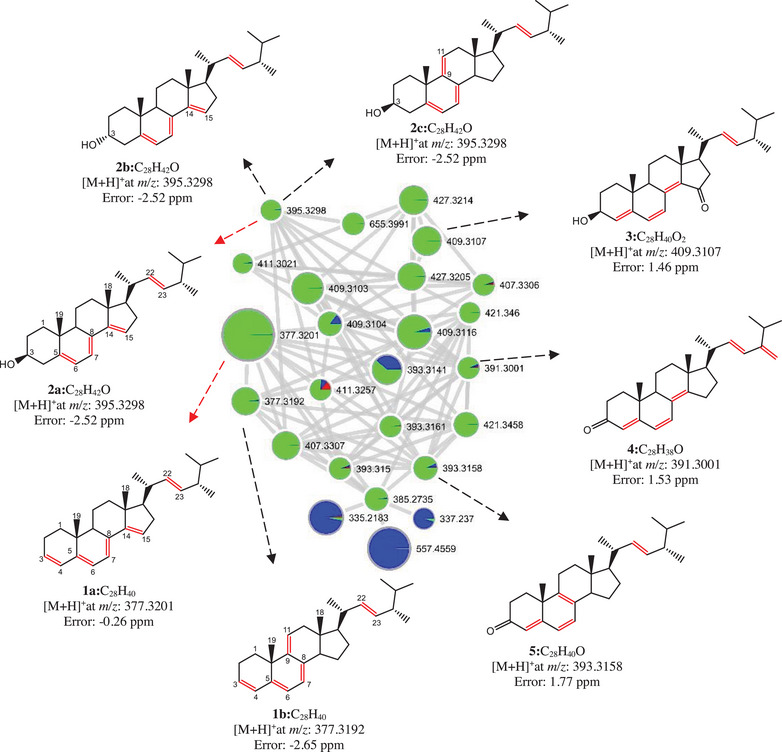
Cluster of steroids annotated in the molecular network obtained from MS/MS data from the fungus *Talaromyces fuscoviridis*. The red nodes indicate the whiteness of the solvent, the blue nodes indicate the culture media only (control), and the green nodes indicate the experiment associated with the fungus, highlighting the isolated steroids.

The substances found with steroidal skeleton were grouped based on the comparison of MS/MS spectra of the five precursor ions ([M + H]^+^) at *m/z* 395.3298, 409.3107, 391.3001, 393.3158, and 377.3192 (Figure 4). All nodes within this cluster exhibited greater daughter ion spectral similarity; thus, they were grouped together. Using this approach, other ions belonging to the same cluster were annotated.

The two ions of nominal mass 377 gave MS/MS spectra annotated for two isomeric polyene steroids, Ergosta‐3(4),7,14,22‐tetraene (*m/z* 377.3201, **1a**, C_28_H_40_) and Ergosta‐3(4),7,9,22‐tetraene (*m/z* 377.3192, **1b**, C_28_H_40_). Three annotations were suggested for de MS/MS spectrum of the precursor ion at *m/z* 395.3298 corresponding to the isomeric compounds Ergosta‐5,7,14,22‐tetraene‐3‐ol (**2a**), Ergosta‐5,7,14,22‐tetraene‐3‐ol (**2b**), and Ergosta‐5,7,9,22‐tetraene‐3‐ol (**2c**), all with a calculated molecular formula of C_28_H_42_O. Three further polyene steroids were annotated with good matches as Gymnasterone‐C (**3**, *m/z* 409.3107, C_28_H_40_O_2_), Ergosta‐4,6,8(14),22,24(28)‐pentaen‐3‐one (**4**, *m/z*391.3001, C_28_H_38_O), and Ergosta‐4,6,8(14),22‐tetraen‐3‐one (**5**, *m/z* 393.3158, C_28_H_40_O).

The evaluation of the steroid cluster drawn based on MS/MS spectral profile and their resemblance to each other (molecular networking) has shown to be a potential tool for the analysis of distribution, annotation, and sometimes identification of different metabolites that may be involved in the adaptation and development processes of various microorganisms. However, our results shown in Figure 2 form a good example of the care needed for these interpretations. The steroids **3**, **4**, and **5** were annotated with very good matches, and they are reported in the literature as molecules identified by NMR data ([27, 28, 29], respectively), suggesting that their molecular structures are correctly assigned. On the other hand, the annotated structures for the steroids **1** (a and b) and **2** (a, b, and c) need further confirmation. These steroids differ from each other only by the position of one of the three C–C double bonds in the tetracyclic ring system, being at Δ^14,15^ for **1a**, **2a**, and **2b** and Δ^9,11^ for **1b** and **2c**. Most of the reports found for Δ^9,11^ steroids describe only mass spectral data obtained by GC–MS [30, 31, 32, 33, 34], and the Δ^14,15^ **1a** contains no spectroscopic data described. Thus, in order to confirm these molecular annotations, the steroids **1** and **2** were targeted for isolation in preparative scale chromatographic procedures.

### 3.3 Isolation of Compounds and Structural Analysis

The fractions rich in polyene steroids were subjected to preparative‐scale separation by HPLC, resulting in two major compounds. Their structures were suggested on the basis of 1D and 2D NMR and HRMS spectroscopic data.

The less polar steroid **1** had its molecular formula established as C_28_H_40_ ([M + H]^+^at *m/z* 377.3201) (Figure S1), a hydrocarbon, whereas steroid **2** was an alcohol with calculated formula C_28_H_42_O ([M + H]^+^at *m/z* 395.3298) (Figure S2).

The ^1^H NMR spectrum obtained for **1** (Figure S3) contains six signals for methyl groups at ca.  *δ* 0.70–1.15, being them four doublets and two singlets, showing great similarity with some signals present in the spectrum of ergosterol [35], a steroid commonly found in fungi. The spectrum also contains a 2*H* multiplet at  *δ* 5.15–5.27, identical to the signals observed for H‐22 and H‐23 of ergosterol, and other five olefinic ^1^H signals at *δ* 5.60–6.20.

The broadband decoupled (Figure S4) and DEPT135 (Figure S5) ^13^C NMR spectra of **1** also corroborate the polyunsaturated hydrocarbon with ergostane skeleton, showing signals for 10 *sp* ^2^ hybridized carbon atoms, being three quaternary and seven methynic (C─H). Moreover, it was not detected any signal that could be ascribed to ^13^C atoms bearing oxygen.

Among the olefinic C─H hydrogens of **1**, there is a pair of doublets at *δ* 5.66 and 6.20 (*J* = 8 Hz, *cis*‐coupled H‐3 and H‐4) in the ^1^H NMR that allowed the positioning of one C─C double bound at ring‐A. The UV spectrum of steroid **1** contains an absorption at *λ*_max_ 319 nm (in MeOH). In ergosterol, the diene at C‐5 and C‐7 produces UV absorption at *λ*_max_ 260 nm [36]. Therefore, the steroid **1** probably contains an extended conjugation of the double bonds at the tetracyclic ring system. The fourth double bound could be positioned at Δ^14,15^ as in **1a** or Δ^9,11^ as in **1b**. There is a correlation in the HMBC 2D spectrum (Figure S6) of **1** between the methyl group CH_3_‐19 (*δ* 0.92, *s*) and the quaternary olefinic carbon ^13^C‐14 (*δ*148.4, *s*) that allows the positioning of this double C─C at Δ^14,15^.

This information was complemented by the HSQC and COSY spectra (Figures S7 and S8). Therefore, the structure of **1** was established as Ergosta‐3(4),5(6),7(8),14(15),22(23)‐pentaene (**1a**).

The elemental composition of steroids **1** and **2** differs by two hydrogens and one oxygen atom (C_28_H_42_O and C_28_H_40_ for **2** and **1**, respectively), which is equivalent to a molecule of water (H_2_O). The ^1^H NMR spectrum of **2** (Figure S9) contains a multiplet at *δ* 3.52–3.72, which is characteristic of H‐3, a carbinolic hydrogen, in ergostane steroids. Thus, probably steroid **2** is equivalent to steroid **1** that had its double‐bound C‐3(4) transformed into alcohol by water addition reaction, forming the Ergosta‐5(6),7(8),14(15),22(23)‐tetraene‐3‐ol steroid (**2a**). All the NMR data acquired for **2a** agrees with those reported in the literature [37] for 14‐dehydroergosterol (14‐DHE), including the HMBC correlation for the methylic hydrogens CH_3_‐19 with the carbon C‐14, which is decisive data for ascribing it as a Δ^14,15^steroid.

When recently isolated, steroid **2a** is detected as a single peak in the chromatogram by UHPLC–DAD and shows UV absorption spectrum very similar to that of **1a**, with *λ*_max_ at 317 nm (Figure S10). This was considered unexpected data, because steroid **1a** is a conjugated pentaene, showing *λ*_max_ at 319 nm, and **2a** contains one double bound less in this p‐system but absorbs at very similar *λ*_max_ (319 nm). This may indicate that double‐bound  Δ^3,4^ in steroid **1a** does not effectively participate in the extended conjugation with the other six *sp* ^2^ carbons in the tetracyclic ring system, and somehow it reacts with water, forming **2a**.

The ^1^H NMR of **2a** (Figure S11) also shows a less intense multiplet at *δ* 4.25–4.38, which is very similar to the above‐mentioned signal for H‐3 in ergostane. The major H‐3 signal at  *δ* 3.52–3.72 was ascribed to a 3β‐OH polyene steroid (**2a**) and the minor one to the 3β‐OH **2b**. Two signals for carbon‐bearing hydroxyl groups in the ^13^C NMR spectrum at *δ* 70.6 (CH) and 68.5 (CH) confirm that **2** is transformed into a mixture of **2a** and **2b** (Figure S12), epimeric polyene steroids, the major one being the Ergosta‐5(6),7(8),14(15),22(23)‐tetraene‐3β‐ol steroid (**2a**). The structure of 2a was further confirmed by SCXRD, providing unambiguous assignment of the hydroxyl position and stereochemistry.

### 3.4 X‐Ray Crystallographic Analysis of Steroid 2a

The positioning of the C─C double bond at Δ^9,11^ for the steroid **2c** (9‐DHE) was established by crystallographic studies reported in the literature [38]. Although the 14‐DHE (**2a**) was studied by 1D and 2D NMR spectroscopy [37], there is no x‐ray analysis of its crystalline structure published. Then it appeared interesting to confirm the steroidal structural assignment from NMR analysis and to figure out how the steroid is organized in crystalline form. The steroid **2a** formed an organized white solid visible as fine needles after slow evaporation of a methanolic solution at room temperature. The structure of this polyene steroid was evaluated by SCXRD, which revealed that it was crystallized in the monoclinic noncentrosymmetric space group P2_1_, with one molecule per asymmetric unit (Table S1).

**FIGURE 5:**
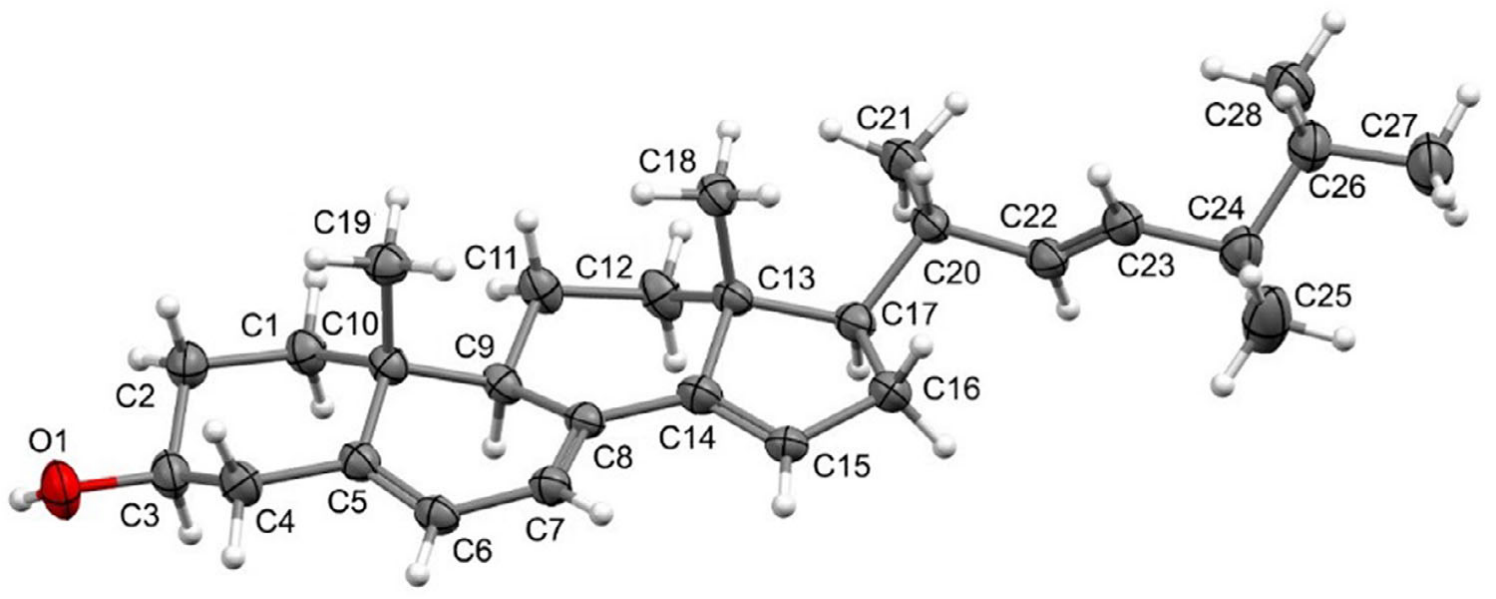
ORTEP type illustration of the asymmetric unit of the steroid 2. Thermal ellipsoids are represented with 50% probability.

An ORTEP drawing for the resolved structure is given below (Figure 5). Thus, the ergostane steroidal skeleton with a triene in the tetracyclic ring system, being one of the double bond at Δ^14,15^, and an additional double bond at the side chain, is confirmed for steroid **2a**.

The SCXRD analysis showed the presence of a hydroxyl group at carbon C‐2 in steroid **2a**. The presence of four C–C double bonds in the structure was also observed, with the C‐5–C‐6, C‐7–C‐8, C‐14–C‐15, and C‐22–C‐23 bonds showing bond lengths in the 1.327–1.347 Å range, as expected for double bonds between two carbon atoms. Table 1 summarizes selected bond lengths and angles for the steroid 2a.

**TABLE 1:** Selected bond lengths and angles for steroid 2a.

Bond lengths (Å)		Bond angles(°)	
C3–O1	1.432(3)	O1–C3–C4	106.9(2)
C5–C6	1.335(3)	C4–C5–C6	121.2(2)
C6–C7	1.450(3)	C7–C8–C14	121.7(2)
C7–C8	1.347(3)	C8–C14–C15	128.8(2)
C14–C15	1.344(3)	C8–C9–C11	114.2(2)
C22–C23	1.327(4)	C20–C22–C23	124.4(2)

The cellular parameters reduced to *a* = 14.8904(3) Å, *b* = 5.79970(10) Å, *c* = 15.0162(3) Å, *α* = 90°, *β* = 111.711(2), *γ* = 90°. The unit cell of this steroid had a crystal unit cell volume of 1204.80(4) Å^3^, with a density of 1088 g/cm^3^, in accordance with that calculated from the unit cell that presented the formula C_28_H_42_O.

Compared to the structure of steroid 9‐DHE [38], which shares the same molecular formula as steroid **2a**, it is observed that the cell parameters are significantly reduced due to the shift in the position of the double bond. This shift would cause variations in the electron density at the C‐9 and C‐11 carbon positions, influencing molecular interactions and, consequently, the crystal packing and physicochemical properties.

This steroid has seven stereocenters, with the atom C‐3 in *S* configuration, whereas the atoms C‐9, C‐10, C‐13, C‐17, C‐20, and C‐24 are *R*‐stereoisomers. As verified in the NMR analyses, steroid **2a** is not stereochemically pure, which resulted in an inversion twin for the crystal of the steroid with two components in a 4:1 ratio and in a Flack parameter of 0.2(5). Furthermore, the SCXRD data agree with the results obtained from the spectroscopic analyses.

The crystal structure of the steroid **2a** is stabilized by O1–H1⋯O1′ hydrogen bonds (dH1⋯O1′ = 2.24 Å and <O1–H1⋯O1′ = 163.6°), which enables the organization of the asymmetric units in a one‐dimensional chain along the direction, as shown in Figure S13.

### 3.5 Influence of Manganese Cation on the Production of Polyenic Steroids

To reduce the dataset dimensionality while retaining maximal relevant information, principal component analysis (PCA) was applied to evaluate the production profiles of steroids in cultures grown with and without the inclusion of the manganese cation (Mn^2+^) in the medium.

PCA was employed for unsupervised data visualization and to identify patterns of clustering, sample distribution, and variable contribution to the total variance. The analysis revealed that the majority of the total variance was efficiently captured by two principal components (PCs). The score plot (Figure S14) shows that the first (PC1) and second (PC2) components collectively accounted for 94.7% of the data variability, highlighting distinct separation patterns in the metabolic profiles across the different experimental conditions.

Analysis of the loadings plot was crucial for determining the influence of individual metabolites (variables) on sample differentiation. The variable clustering observed reinforced the chemical equivalence of the compounds isolated from the different cultures. Critically, the PCA results confirmed that the presence of the inducer was responsible for a change in the relative proportion of the steroids, suggesting a direct regulatory impact of manganese on secondary metabolite biosynthesis.

**FIGURE 6:**
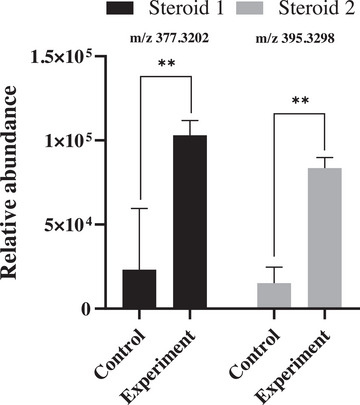
Relative abundance plot of steroids from the fungus *Talaromyces fuscoviridis* in a culture medium with and without manganese. Bars represent the mean $\pm$ standard deviation. ** indicates a statistically significant difference p < 0.05, between the Control and Experiment groups, demonstrating that the presence of manganese strongly affects the concentration of both steroids.

This observation aligns with the well‐established principle that metal cations can act as regulatory factors capable of modulating microbial metabolic pathways [39, 40]. In the present study, cultivation of *Talaromyces fuscoviridis* in the presence of manganese resulted in a marked increase in the relative concentration of the target metabolites (Figure 6), thereby corroborating the PCA results and confirming the direct regulatory role of this metal ion in fungal secondary metabolism.

Manganese ion (Mn^2+^) serves as an essential cofactor for numerous enzymes, including those integral to steroid biosynthesis. Its presence can directly activate key enzymes responsible for precursor conversion and enhance the activity of the mevalonate pathway, which is central to isoprenoid and sterol synthesis.

Specifically, they may regulate enzymes such as cytochrome P450 monooxygenases, which play a critical role in the structural modification and diversification of sterols. Consequently, the observed increase in steroid production is likely attributable to the activation of these metalloenzymes and the resulting enhanced flux of metabolic intermediates in the presence of this cation. This suggests that Mn^2+^ acts as a metabolic inductor, directly influencing the fungal secondary metabolism.

### 3.6 Evaluation of the Chromatographic Method and the Antioxidant Potential of Isolated Compounds

**FIGURE 7:**
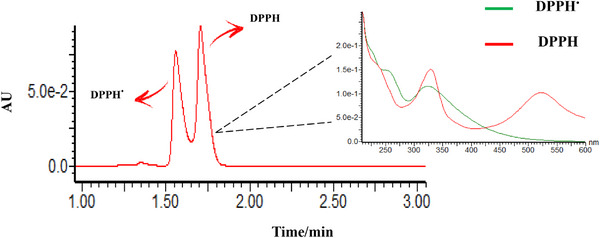
Chromatographic and spectroscopic characterization of DPPH and DPPH^•^ radicals. UHPLC chromatogram showing the separation of DPPH^•^ (1.45 min) and DPPH (1.85 min) peaks obtained under isocratic elution with 70% acetonitrile and 30% water. The inset displays the corresponding UV–Vis spectra of DPPH^•^ (green line) and DPPH (red line), highlighting the characteristic absorption decrease at 517 nm after radical reduction. DPPH, 2,2‐diphenyl‐1‐picrylhydrazyl.

Free radicals are very unstable chemical species with a great capacity for cell damage. In biological systems, they are mainly hydroxyl (OH^•^), superoxide (O_2_^•−^), peroxyl (ROO^•^), and lipid peroxyl (LOO^•^), and some nitrogenous species [41]. Thus, it is interesting to find antioxidant substances capable of trapping these aggressive chemical entities, being useful as cell protectors. One of the most common methods to evaluate the antioxidant capacity of cell protector candidates measures the extension of trapping free radicals of DPPH [42, 43]. DPPH free radicals (DPP^•^) exhibit a maximum absorption at 517 nm. As its odd electron becomes paired in the presence of an H^•^ donor, it transforms into the stable form of its reduced pair (DPPH), which absorbs at a lower wavelength, in the range of 330 nm (Figure 7) [43, 44]. Therefore, the absorption at 517 nm with and without putative H^•^ donors reflects the ratio [DPP^•^]/[DPPH] and can be associated to antioxidant activity.

Although the direct spectrophotometric method for evaluating DPP^•^ radicals is widely used to assess antioxidant activity, it may present some limitations when applied to matrices that exhibit absorption interferences. This is particularly the case for polyene steroids, which absorb around 320 nm and therefore overlap with the characteristic absorption of DPPH at 330 nm. Hence, various methodological refinements have been proposed to overcome these limitations [23, 45]. Chromatographic separations using HPLC have proven to be a more specific and sensitive analytical approach to evaluate the DPP^•^ scavenging activity.

During the present study, an even faster and sensitive method using an ultra‐high performance liquid chromatography coupled with diode‐array detection (UHPLC–DAD) was developed to evaluate the free radical scavenging capacity of the polyene steroids. Although most of the literature methods use polyphenols such as flavonoids as reference antioxidants, our method used β‐carotene as a reference compound, because the steroids possess a lipophilic characteristic. After the reaction time between the antioxidant candidate and the DPP^•^, the reactional mixture was injected in the UHPLC system, resulting in two chromatographically resolved peaks corresponding to DPPH and DPP^•^ within 1.5–2.0 min (Figure 7). The success of this method is also improved by the use of a mobile phase predominantly composed of organic solvents without H^•^ donors to ensure a level of stability of DPP^•^ radical. Therefore, the mobile phase uses acetonitrile/water gradient and must be out of methanol because it also reacts with the DPP^•^. Other factors such as higher water content in the mobile phase, addition of acid to the mobile phase, and the initial concentration of DPPH radicals are also considered important factors that may influence the stability of this radical [23, 43, 44]. The adaptation of the DPPH radical assay to the UHPLC separation method was considered a satisfactory method of choice for this study, which was found fast and reliable.

**FIGURE 8:**
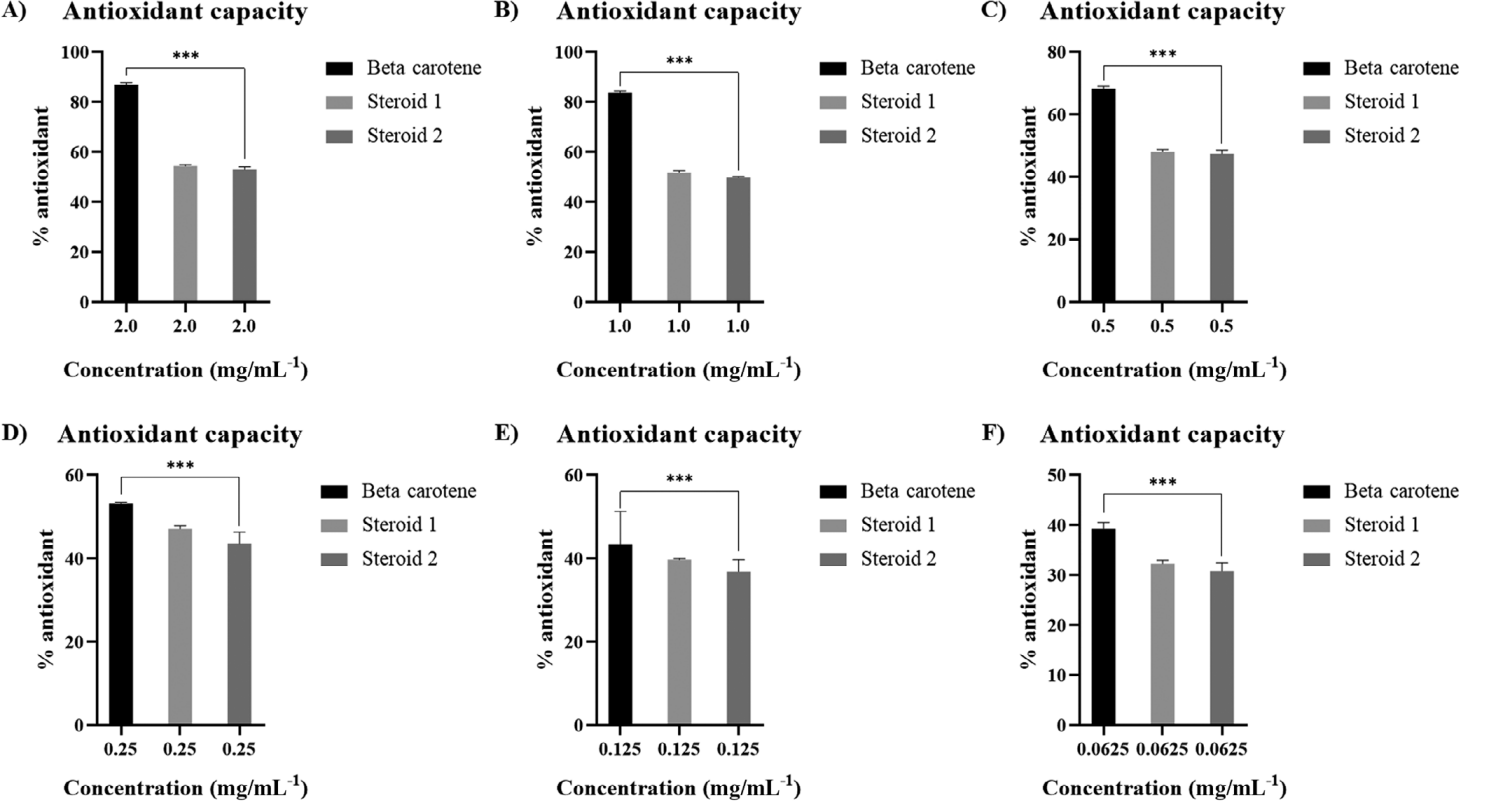
Antioxidant potential of steroids **1a** and **2a** and the  β‐carotene standard against DPPH free radicals with 95% sensitivity. The concentrations used of the compounds were (A) 2.0 mg/mL, (B) 1.0 mg/mL, (C) 0.5 mg/mL, (D) 0.25 mg/mL, (E) 0.125 mg/mL, and (F) 0.0625 mg/mL. Bars represent the mean $\pm$ standard deviation. ** indicates a statistically significant difference p < 0.05.

Steroids **1a** and **2a** exhibited very similar DPPH free radical scavenging activity in the concentration range of 2.0 to 0.0625 mg/mL, with inhibition percentage exceeding 55% at higher concentrations and drops to ca. 30% at lower concentrations (=6.25 ng/mL) (Figure 8A–F). This activity may be related to the structural characteristic of these compounds when compared to other similar structures such as ergosterol. Steroid like compounds also exhibit certain activity against these radicals [43, 45, 46].

The reference compound  β‐carotene inhibits ca. 80% of DPP^•^ radical at higher concentrations. It is interesting to note that the steroids **1** and **2** show almost the same radical scavenger capacity as the β‐carotene at lowers concentrations (Figure 8D–F), over 80%. To date, there have been no reports in the literature regarding the antioxidant activity of these compounds associated with the fungus *Talaromyces fuscoviridis*, although other studies suggest that fungi belonging to this genus possess different biological properties [47].

The majority of natural antioxidants are recognized as polar, hydrophilic polyphenolic substances [48]. The high efficacy of polyphenols stems from the facile homolytic cleavage of their O–H bonds, which donates a hydrogen radical (H^•^) to scavenge free radical species. The resultant phenoxide radical is significantly stabilized by the extended aromatic system [49]. Consequently, polyphenols are typically characterized as strong, fast, and often non‐specific antioxidants.

In contrast, polyene compounds, such as  β‐carotene (used as the reference compound in this work) and the tetraene steroids, do not exhibit phenolic hydroxyl groups. Their antioxidant action is primarily attributed to the allylic hydrogens adjacent to the conjugated double bonds within the polyene skeleton [50]. This antioxidant activity is mainly attributed to the allylic hydrogens adjacent to the conjugated double bonds in the polyene steroid skeleton. These hydrogens can act as H^•^ donors, stabilizing reactive oxygen species (ROS) through radical abstraction. The extensive π‐conjugation across the polyene framework facilitates delocalization of the resulting unpaired electron, thereby stabilizing the radical species formed after hydrogen donation.

This mechanism is analogous to that observed in phenolic antioxidants but differs due to the absence of hydroxyl groups on aromatic rings and the lipophilic nature of the steroid nucleus, which enhances radical scavenging in apolar environments such as cell membranes. Additionally, the presence of a hydroxyl group at C‐3 in steroid 2a may further contribute to radical stabilization through intramolecular hydrogen bonding, potentially enhancing antioxidant efficiency.

Allylic C─H bonds are slightly stronger than the O─H bonds found in phenolic antioxidants. Consequently, carotenoids and polyene steroids generally display weaker overall antioxidant capacity but exhibit higher specificity, primarily due to their pronounced lipophilicity. A plausible hypothesis arising from this observation is that polyphenols function predominantly as extracellular antioxidants, whereas lipophilic steroids and carotenes may protect cell membranes by modulating or neutralizing invading oxidant species.

Many fungal species demonstrate a remarkable capacity to thrive in challenging environments, such as soils contaminated with heavy metals or habitats characterized by significant fluctuations in humidity and temperature. These extreme conditions, including high heavy metal concentrations, elevated UV radiation, and high temperatures, promote the excessive generation of ROS. Oxidative stress resulting from ROS exposure can destabilize cellular membranes and degrade biomolecules vital for fungal development. Understanding the adaptive mechanisms of these microorganisms to such selective pressures has been a major research focus [51, 52, 53]. The production of antioxidants is hypothesized to be a key fungal survival mechanism, as these compounds neutralize free radicals, mitigating cellular damage and enabling persistence. Furthermore, in nutrient‐limited environments, these compounds may also assist in the absorption of essential nutrients, thereby optimizing fungal growth and reproduction. Consequently, polyene steroids, along with other yet‐unidentified metabolites, may collectively form an antioxidant arsenal that contributes significantly to the survival of the fungus *Talaromyces fuscoviridis* in its ecological niche.

## 4 Conclusion

The results demonstrate that *Talaromyces fuscoviridis* is a promising source of compounds exhibiting antioxidant activity. Specifically, the isolated steroids effectively reduced DPPH free radicals. This observed activity is directly correlated with the chemical structure and concentration of the compounds, highlighting their potential as protective agents against oxidative stress. The use of ultra‐high‐performance liquid chromatography coupled with diode‐array detection (UHPLC–DAD) proved to be an effective, sensitive, and reproducible approach for monitoring this bioactivity. The integration of chromatographic data, molecular networking, and biological assays facilitated the precise identification of bioactive substances with prospective utility as natural antioxidants. These findings underscore the critical role of advanced analytical strategies in the bioprospecting of fungal metabolites for subsequent application in the pharmaceutical, food, and biotechnological industries.

## Author Contributions

**Mauricio Augusto Pinto Moreno da Silva Alves**: data curation, formal analysis, investigation, methodology, writing – original draft, writing – review and editing. **Alef dos Santos**: conceptualization, data curation, formal analysis, investigation, methodology, visualization, writing – original draft, writing – review and editing. **Eduardo Jorge Pilau**: formal analysis, methodology, writing – review and editing. **Pedro Henrique de Oliveira Santiago**: formal analysis, investigation, writing – original draft. **Javier Alcides Ellena**: formal analysis, methodology, visualization, writing – review and editing. **Marilene Nunes Oliveira**: investigation, resources, writing – review and editing. **Edson Rodrigues Filho**: conceptualization, funding acquisition, project administration, writing – original draft, writing – review and editing.

## Funding

The authors would like to thank the funding from the São Paulo State Foundation (FAPESP—2017/15850‐0 and 2019/04900‐2), National Council for Technological and Scientific Development (CNPq—312505/2021‐3 and 405940/2021‐1), and the Coordination for the Improvement of Higher Education (CAPES).

## Conflicts of Interest

The authors declare no conflicts of interest.

## Supporting information




**Supporting File 1**: cbdv70690‐sup‐0001‐SuppMat.docx

## Data Availability

The annotated genome sequence of *Talaromyces fuscoviridis* has been deposited in the GenBank database. The GNPS workflows for LC–HRMS data acquired in positive ionization mode are accessible at https://gnps2.org/result?task = 1ffee75fe0d40a6be53b479cf4f7719&viewname=librarymatches&resultdisplay_type = task. Deposition Number(s) “https://www.ccdc.cam.ac.uk/services/structures?id = doi:10.1002/###.20230928EF01_auto” contain(s) the supplementary crystallographic data for this article. These data are provided free of charge by the joint Cambridge Crystallographic Data Centre and Fachinformationszentrum Karlsruhe “http://www.ccdc.cam.ac.uk/structures”Access Structures service</url>. Additional data supporting the findings of this study are available from the corresponding author upon reasonable request.
