# Supplementary material for: Manganese‐Induced Production of Antioxidant Polyene Steroids in the Extremophile Fungus Talaromyces fuscoviridis Isolated From Copper‐Mine Soil
Source: Chem Biodivers. 2025 Nov 21;23(1):e01816. doi: 10.1002/cbdv.202501816 (PMC12761365; doi:10.1002/cbdv.202501816)
Supplement: Supplementary file 1 — Supporting File 1: cbdv70690‐sup‐0001‐SuppMat.docx [file CBDV-23-e01816-s001.docx]

**Contents:**

**Table 1-SM.** Crystallographic parameters of steroid 2a.

**Figure 1-SM.** Mass spectrum of steroid 1a (HRMS spectrum ESI ionization source).

**Figure 2-SM.** Mass spectrum of steroid 2a (HRMS spectrum ESI ionization source).

**Figure 3-SM.** ^1^H NMR spectrum of steroid 1a (400 MHz, CDCl_3_).

**Figure 4-SM.** ^13^C NMR spectrum of steroid 1a (400 MHz, CDCl_3_).

**Figure 5-SM.** DEPT NMR spectrum of steroid 1a (400 MHz, CDCl_3_).

**Figure 6-SM.** HMBC NMR spectrum of steroid 1a (400 MHz, CDCl_3_).

**Figure 7-SM.** HSQC NMR spectrum of steroid 1a (400 MHz, CDCl_3_).

**Figure 8-SM.** COSY NMR spectrum of steroid 1a (400 MHz, CDCl_3_).

**Figure 7-SM.** ^1^H NMR spectrum of steroid 2a (400 MHz, CDCl_3_).

**Figure 8-SM.** ^1^H spectrum of steroid 2a, which showed the characteristic of an epimer with a difference in the configuration of carbon 3.

**Figure 9-SM.** ^13^C NMR spectrum of steroid 2a (400 MHz, CDCl_3_).

**Figure 10-SM.** Chemical structure and ultraviolet absorption of steroids 1a and 2a.

**Figure 11-SM.** ^1^H spectrum of steroid 2a, which showed the characteristic of an epimer with a difference in the configuration of carbon 3.

**Figure 12-SM.** ^13^C NMR spectrum of steroid 2a (400 MHz, CDCl_3_).

**Figure 13-SM.** One-dimensional chain formed for the steroid **2** with the intermolecular O1–H1···O1’ interactions, viewed in the [001] direction.

**Figure 14-SM.** PCA score plot of PC1 and PC2, using chromatographic data processed alongside the cultivation data of the fungus *Talaromyces fuscoviridis* in a culture medium with and without manganese.

| **Description** | **Steroid 2** |
| --- | --- |
| Chemical formula | C_28_H_42_O |
| Molar mass (g mol^-1^) | 394.61 |
| Crystal system | Monoclinic |
| Space group | P2_1_ |
| *a* (Å) | 14.8904(3) |
| *b* (Å) | 5.79970(10) |
| *c* (Å) | 15.0162(3) |
| β (o) | 111.711(2) |
| V (Å^3^) | 1204.80(4) |
| Z | 2 |
| Density /g cm^-3^ | 1.088 |
| θ­min, θ­max /^o^ | 6.336, 158.918 |
| Index ranges | –18 ≤ *h* ≤ 18 |
|  | –7 ≤ *k* ≤ 7 |
|  | –18 ≤ *l* ≤ 14 |
| F(000) | 436.0 |
| μ /mm^-1^ | 0.473 |
| Absorption correction | Multi-scan |
| Max./min. transmission | 1.000/0.781 |
| Measured reflections | 29309 |
| Independent reflections / R_int_ | 5170/0.0486 |
| Refined parameters | 270 |
| Final R indexes [I≥2σ(I)] | R_1_= 0.0445/wR_2_= 0.1113 |
| Final R indexes [all data] | R_1_= 0.0472/wR_2_= 0.1129 |
| GooF | 1.059 |
| Largest diff. peak and hole (eÅ^-3^) | 0.30/-0.19 |
| Flack parameter | 0.2(5) |

**Table 1-SM.** Crystallographic parameters of steroid 2a.


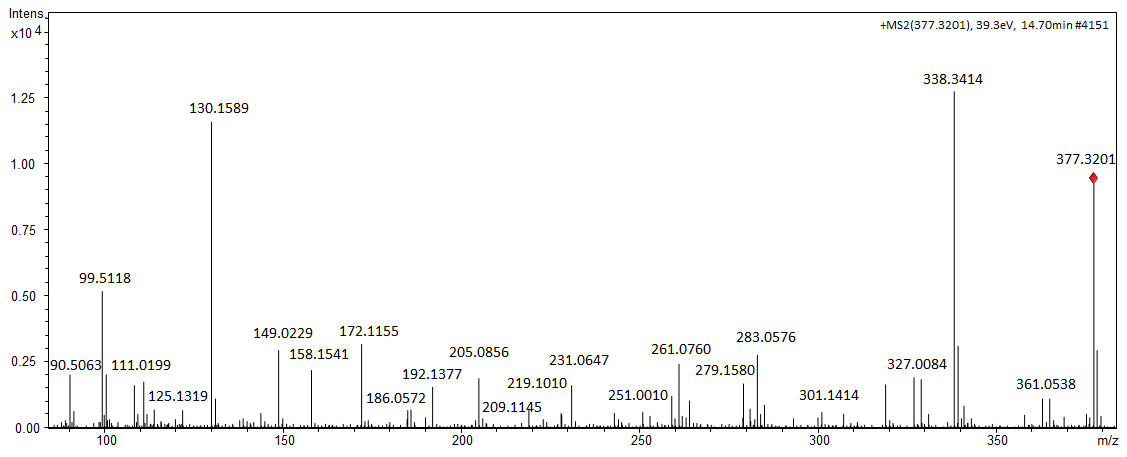


**Figure 1-SM.** Mass spectrum of steroid 1a (HRMS spectrum ESI ionization source).


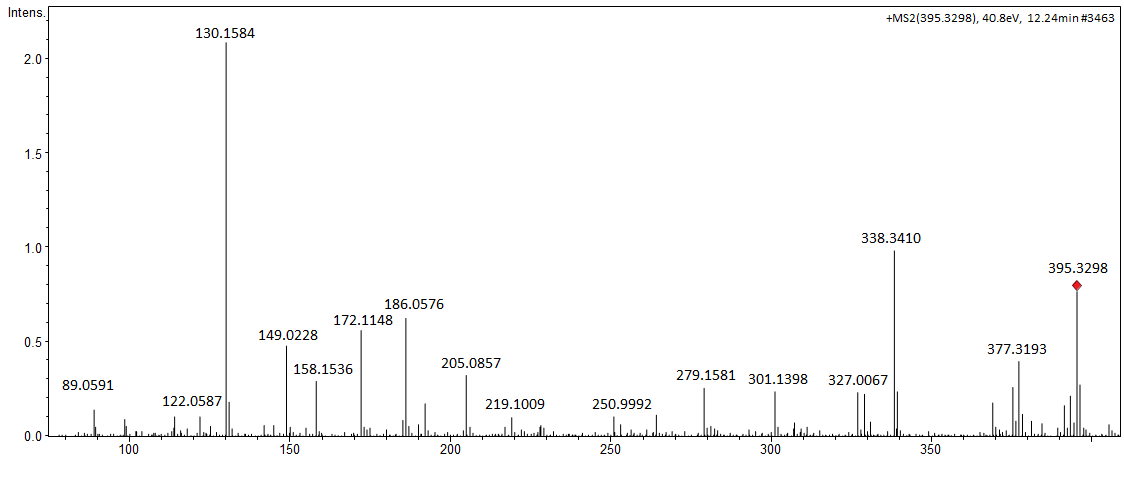


**Figure 2-SM.** Mass spectrum of steroid 2a (HRMS spectrum ESI ionization source).

**Figure 3-SM.** ^1^H NMR spectrum of steroid 1a (400 MHz, CDCl_3_).


**Figure 4-SM.** ^13^C NMR spectrum of steroid 1a (400 MHz, CDCl_3_).

**Figure 5-SM.** DEPT NMR spectrum of steroid 1a (400 MHz, CDCl_3_).

**Figure 6-SM.** HMBC NMR spectrum of steroid 1a (400 MHz, CDCl_3_).

**Figure 7-SM.** HSQC NMR spectrum of steroid 1a (400 MHz, CDCl_3_).


**Figure 8-SM.** COSY NMR spectrum of steroid 1a (400 MHz, CDCl_3_).

**Figure 9-SM.** ^1^H NMR spectrum of steroid 2a (400 MHz, CDCl_3_).

**1a**

**2a**

**Figure 10-SM.** Chemical structure and ultraviolet absorption of steroids 1a and 2a.

**Figure 11-SM.** ^1^H spectrum of steroid 2a, which showed the characteristic of an epimer with a difference in the configuration of carbon 3.

**Figure 12-SM.** ^13^C NMR spectrum of steroid 2a (400 MHz, CDCl_3_).


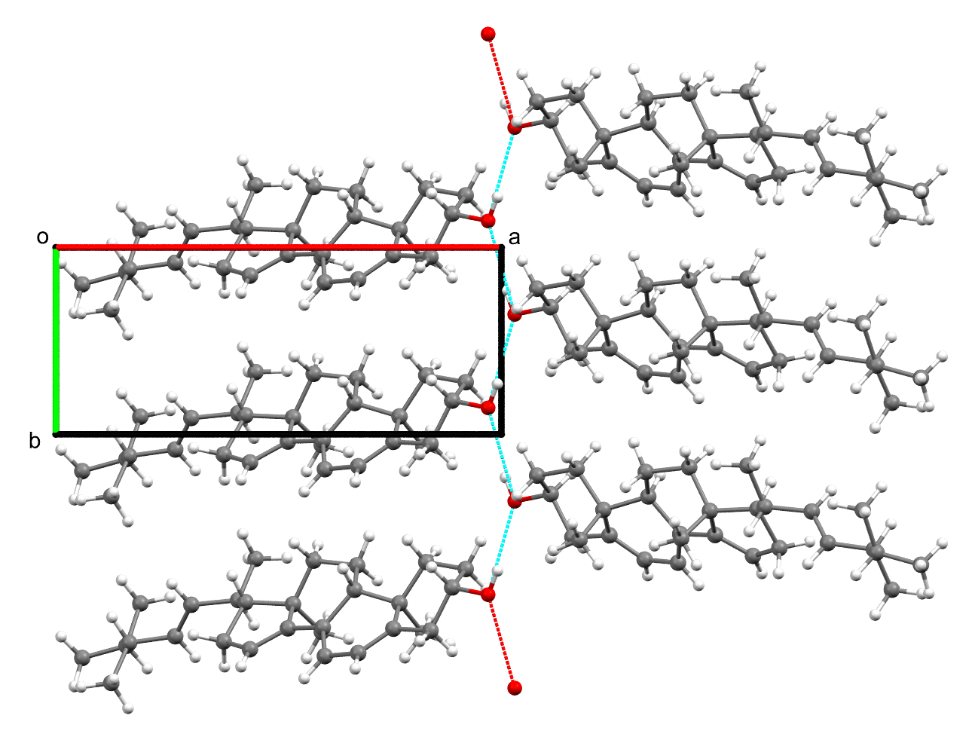


**Figure 13-SM.** One-dimensional chain formed for the steroid **2** with the intermolecular O1–H1···O1’ interactions, viewed in the [001] direction.


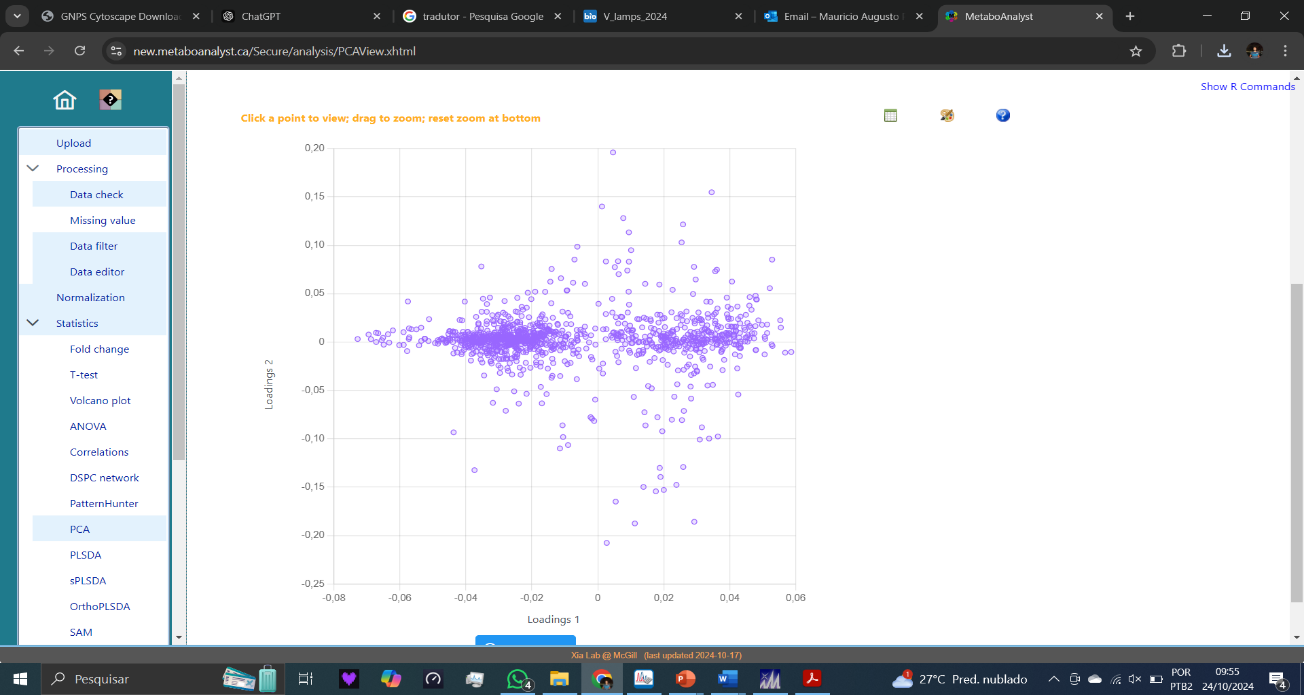

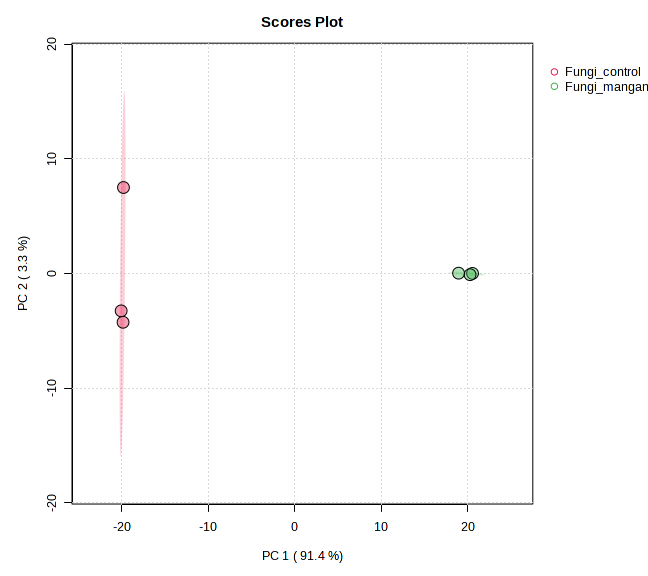


**Rice**

**Rice Mn^2+^**

**Rice**

**Rice Mn^2+^**

**PCA**

**Loadingsplots**

**Figure 14-SM.** PCA score plot of PC1 and PC2, using chromatographic data processed alongside the cultivation data of the fungus *Talaromyces fuscoviridis* in a culture medium with and without manganese.
